# Construction of the first high-density genetic linkage map and QTL mapping of flavonoid and leaf-size related traits in *Epimedium*

**DOI:** 10.1186/s12870-023-04257-0

**Published:** 2023-05-25

**Authors:** Dongyue Yu, Ruoqi Huang, Shuxia Yu, Qiong Liang, Ying Wang, Haishan Dang, Yanjun Zhang

**Affiliations:** 1 Key Laboratory of Aquatic Botany and Watershed Ecology, Wuhan Botanical Garden, Chinese Academy of Sciences, Wuhan, 430074 P.R. China; 2 University of Chinese Academy of Sciences, Beijing, 100049 P.R. China; 3 Key Laboratory of Plant Germplasm Enhancement and Specialty Agriculture, Wuhan Botanical Garden, Chinese Academy of Sciences, Wuhan, 430074 P. R. China; 4 Key Laboratory of South China Agricultural Plant Molecular Analysis and Genetic Improvement, Provincial Key Laboratory of Applied Botany, South China Botanical Garden, Chinese Academy of Sciences, Guangzhou, 510650 P.R. China

**Keywords:** *Epimedium*, Genetic linkage map, Leaf size, Epimedin C

## Abstract

**Background:**

Leaves are the main medicinal organ in *Epimedium* herbs, and leaf flavonoid content is an important criterion of *Epimedium* herbs. However, the underlying genes that regulate leaf size and flavonoid content are unclear, which limits the use of breeding for *Epimedium* development. This study focuses on QTL mapping of flavonoid and leaf-size related traits in *Epimedium*.

**Results:**

We constructed the first high-density genetic map (HDGM) using 109 F1 hybrids of *Epimedium leptorrhizum* and *Epimedium sagittatum* over three years (2019–2021). Using 5,271 single nucleotide polymorphism (SNP) markers, an HDGM with an overall distance of 2,366.07 cM and a mean gap of 0.612 cM was generated by utilizing genotyping by sequencing (GBS) technology. Every year for three years, 46 stable quantitative trait loci (QTLs) for leaf size and flavonoid contents were discovered, including 31 stable loci for Epimedin C (EC), one stable locus for total flavone content (TFC), 12 stable loci for leaf length (LL), and two stable loci for leaf area (LA). For flavonoid content and leaf size, the phenotypic variance explained for these loci varied between 4.00 and 16.80% and 14.95 and 17.34%, respectively.

**Conclusions:**

Forty-six stable QTLs for leaf size and flavonoid content traits were repeatedly detected over three years. The HDGM and stable QTLs are laying the basis for breeding and gene investigation in *Epimedium* and will contribute to accelerating the identification of desirable genotypes for *Epimedium* breeding.

**Supplementary Information:**

The online version contains supplementary material available at 10.1186/s12870-023-04257-0.

## Background

For almost 2,000 years, plants of the genus *Epimedium*, which belongs to the family Berberidaceae, have been used in Chinese medicine to strengthen muscles and bones [1]. Furthermore, *Epimedium* has a variety of biological actions, including anticancer, antioxidant, and antirheumatic effects [2, 3]. Previous studies have reported that most of these medicinal properties are attributed to leaf flavonoid glycosides [4]. In China, the herb *Epimedium*, which is produced from wild resources, is offered using aerial plant parts, primarily dried leaves [4]. Thus, wild *Epimedium* resources have gradually shrunk due to overexploitation in recent decades [5]. Furthermore, the chemical synthesis of flavonoid glycosides was limited by the low rate of recovery [2]. Epimedin C, a derivative of the phenylpropanoid pathway, is the major bioactive constituent of plant secondary metabolites in leaves. It has been recommended as a reference for the quality control of *Epimedium* herba [6, 7]. However, the complex biosynthesis process of secondary metabolites is not completely understood [8]. To date, only some flavonoid biosynthetic process enzymes have been identified, such as EpPF3RT, EpRhS and Ep7GT from *E. pseudowushanense* [2]. The leaf impacts plant development and biomass as the primary organ generated for consumption [9] and is the main medicinal organ in traditional herbals of *Epimedium.* At the molecular level, leaf size is generally coordinated and driven by multiple integrated signals of plant hormone production and signalling [9, 10]. The molecular mechanisms of leaf size have been revealed through a combination of genetic and intense analyses in *Arabidopsis thaliana* [10, 11], *Medicago truncatula* [12], *Lactuca sativa* [13] and *Brassica* [9] plant species. However, directional breeding of *Epimedium* for flavonoid glycoside content and leaf blade size is a substantial obstacle due to a lack of comprehension of the mechanisms of leaf blade generation and the flavonoid glycoside biosynthesis pathway. Therefore, it is essential to investigate the underlying QTLs governing leaf-size and flavonoid content features to develop novel varieties with high medicinal value.

Conventional breeding procedures are based on the phenotypic choice of genotypes from separated offspring generated by hybridization, which are usually limited by intensive labor, cost, and time efficiency [14]. Marker-assisted selection (MAS), a methodology of species selection enabled by molecular markers, has the promise to replace phenotypic selection systems [15, 16]. Based on MAS, we might significantly accelerate the genetic improvement of desired agronomic qualities by selecting plants directly and effectively according to their genotypes [16, 17]. In molecular plant breeding, saturated HDGM employing molecular markers of great genome coverage is essential for MAS of advantageous alleles or traits [18, 16]. Quantitative trait locus (QTL) mapping based on HDGM is a technique used to discover closely affiliated molecular markers and to determine the genetic region linked to targeted phenotypes [19, 20]. Due to their abundance and density across the entire genome, single nucleotide polymorphism (SNP) loci offer an effective method for MAS, HDGM construction, and QTL mapping [21, 19]. Genotyping by sequencing (GBS) produces an enormous number of SNPs that are used to investigate species with large and complex genomes and has been extensively used in HDGM construction and QTL mapping of various species [15, 19, 16] including *Momordica charantia* [22], *Cynodon dactylon Pers* [23], and *Schima superba* [16]. Thus, GBS techniques and QTL analysis associated with target traits could also be effective methods for the MAS of selective breeding for *Epimedium*.

An HDGM is essential for QTL evaluation [16]; however, no HDGM has been constructed for *Epimedium*. Therefore, HDGM is required for the discovery of important genes associated with phenotypes in *Epimedium*. In this study, SNPs were mapped to the *E. sagittatum* reference genome and were subsequently used to construct an *Epimedium* HDGM based on an F1 full-sib mapping family generated from a hybrid between *E. leptorrhizum* and *E. sagittatum* by utilizing SNP loci obtained by double-digest GBS technology. The leaf length (LL), leaf area (LA), Epimedin C (EC) and total flavone content (TFC) of individual plants were determined for three consecutive years. These phenotypic traits were then QTL evaluated to determine relevant SNP markers and potential positions on the HDGM. The development of an HDGM of *Epimedium* and QTL evaluation of economic traits can assist in clarifying the related genetic basis to improve MAS methods and *Epimedium* genetic improvements.

## Results

### Variability characteristics of the four mapping traits in parents and progeny

**Fig. 1:**
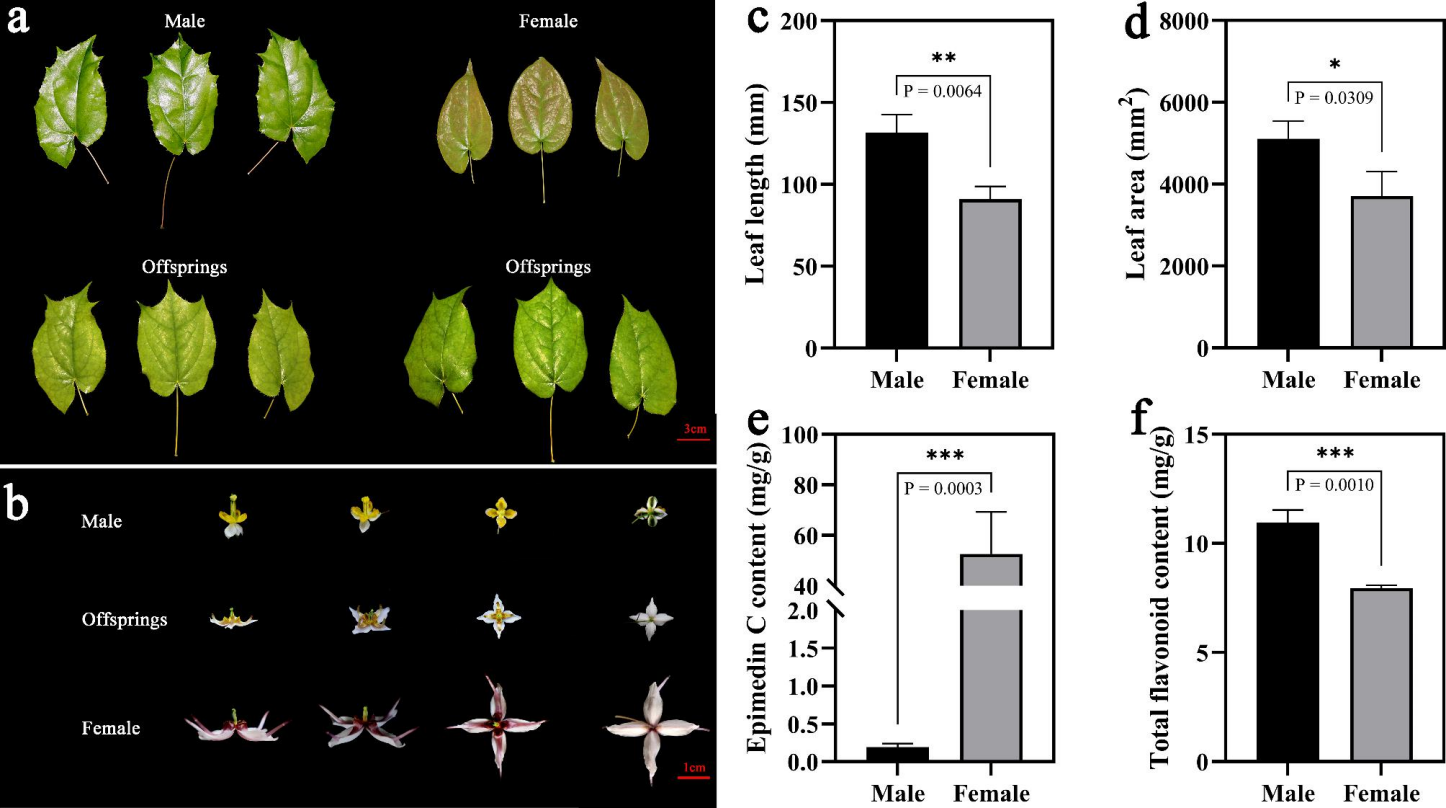
Phenotypic characteristics and flavonoid content of male (*Epimedium sagittatum)* and female (*Epimedium leptorrhizum*) parents. (**a**) Male and female parent plant leaves, (**b**) flowers of the hybrid offspring parent. There were significant differences in leaf length (**c**), leaf area (**d**), Epimedin C content (**e**) and total flavonoid content (**f**) between the parents

The two parents showed unique characteristics in the EC, TFC, LL and LA traits (Fig. 1). Four traits from three separate years were subjected to analysis of variance (ANOVA), and the results indicated no discernible variation across the years (Supplementary Table S1). The skewness and kurtosis values were both less than one, except for the kurtosis values for TFC, which varied from 4.575 (2021) to 17.379 (2020). The descriptive statistical analysis of the four mapping traits showed that the coefficient of variation (CV%) varied from 14.636 (2021) to 17.195 (2019) for EC, from 12.247 (2020) to 19.712 (2019) for TFC, from 17.601 (2021) to 20.367 (2020) for LL, and from 31.637 (2021) to 31.946 (2019) for LA (Supplementary Table S2). Except for TFC, both the frequency distribution histogram and normality test results (Supplementary Table S3) among the three mapping traits for three consecutive years (2019–2021) showed a positive normal distribution. Despite the absence of a significant correlation between the other traits, the correlation analysis showed a significant association between LL and LA (Supplementary Fig. S1). Positively correlated traits (such as LL and LA) showed close relationships across genetic markers due to pleiotropic effects, which might benefit the prediction of candidate genes.

### GBS library sequencing and SNP discovery

GBS sequencing provided 1,404,295,474 150 bp paired end reads and 186,913,974,238 bp clean reads (Supplementary Table S4). For the male and female parents, 106,416,658 (40.65% guanine-cytosine (GC) content with a Q30 of 94.75%) and 100,046,576 (40.65% guanine-cytosine (GC) content with a Q30 of 94.75%) reads were both aligned to the *E. sagittatum* genome, respectively. The F1 population had an average of 10,989,287 clean reads, with a range of 6,179,886 to 33,063,778. In addition, the average GC content of the 109 descendant sequences was 41.15%, and the Q30 score was 94.77% (Supplementary Table S4). In these reads, a total of 46,594 SNPs were obtained after comparison with the reference genome and filtering of SNPs. Given that the two parents were cross-pollinator lineages, 14,115 SNP markers from genotypes lm×ll (13,749), hk×hk (310), and ef×eg, (56) were employed to generate the HDGM (Supplementary Table S5).

### Developing an HDGM based on the ***E. sagittatum*** reference genome

**Fig. 2:**
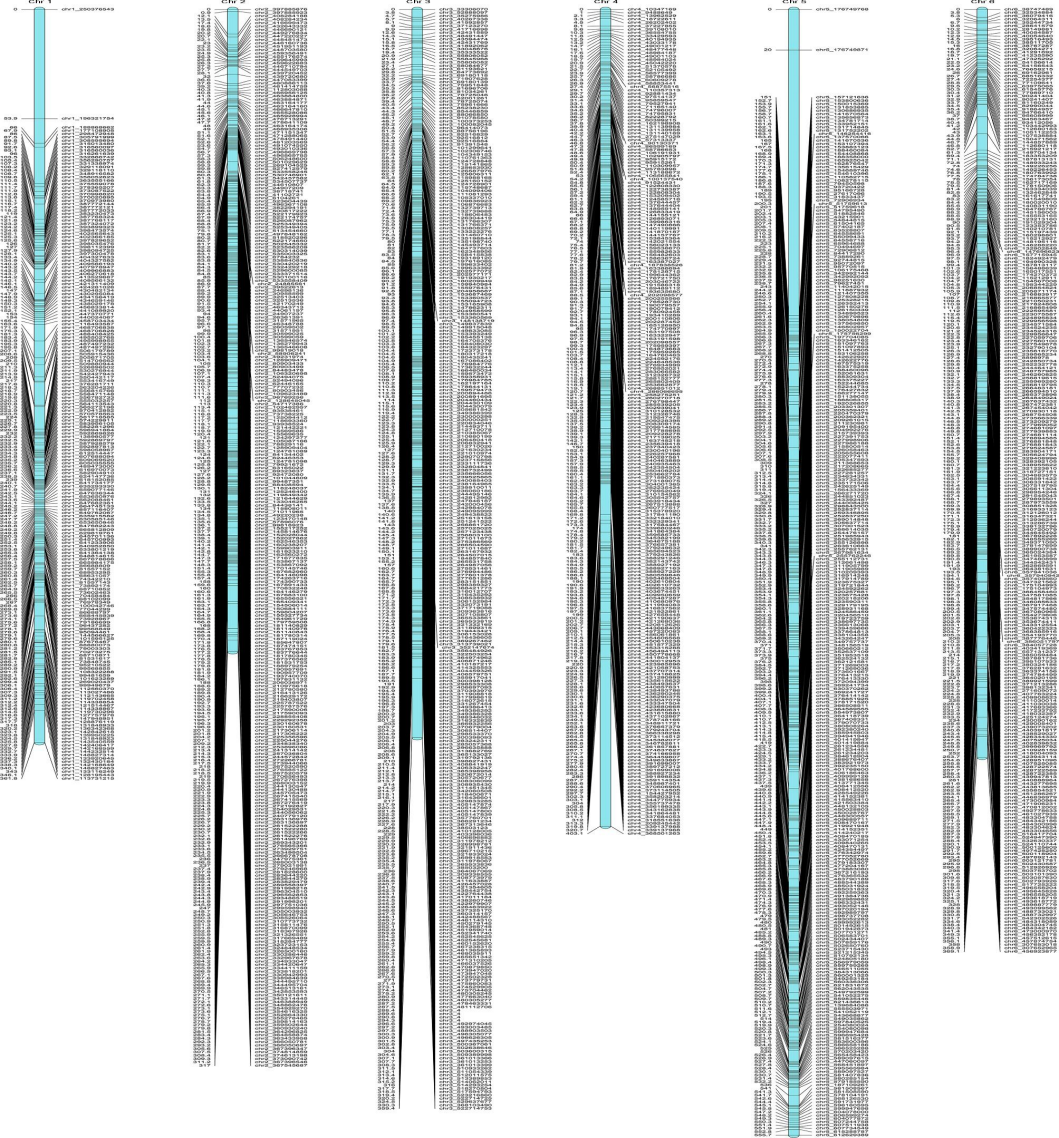
Genetic map of *Epimedium* based on 5,271 SNPs. The marker distribution is shown by the black bars on each linkage group

According to their location on the *Epimedium sagittatum* reference genome, 14,115 SNP markers were successfully allocated to six linkage groups (LGs). The paternal map comprised 5,269 markers distributed among six LGs, with an overall genetic length of 3246.91 cM and a mean marker gap distance of 0.84 cM. LG had a mean length of 541.15 cM, varying between 457.27 cM (LG2) and 785.62 cM (LG5) (Supplementary Table S6 and Fig. S2). On the maternal map, 392 markers were positioned in six LGs, with an overall genetic length of 985.82 cM and LG length varying from 131.02 cM (LG2) to 190.69 cM (LG4) (Supplementary Table S7 and Fig. S3). The consensus HDGM, which was generated by 5,271 SNPs, had a length of 2,366.07 cM and an average intermarker distance of 0.612 cM (Table 1; Fig. 2). The results showed that the most saturated chromosome was LG2, which contained 1,379 SNPs covering 317.03 cM with a mean intermarker distance of 0.23 cM. The longest LG5 included 442 markers, a total length of 555.66 cM, and an average gap of 1.30 cM, while the smallest LG6 had 365 markers, an overall distance of 369.142 cM, and a mean gap of 1.00 cM. The less than five cM gaps of a single LG ranged from 97.28% (LG5) to 99.69% (LG3). The longest gap in the consensus HDGM was 130.98 cM, which was present on LG5 (Table 1; Fig. 2).

**Table 1:** Description of the basic characteristics of the 6 linkage groups

Linkage/n Group ID	Total/n Marker	Total/n Distance (cM)	Average/n Distance (cM)	Max/n Gap (cM)	Gap/n < 5 cM (%)	Spearman/n correlation coefficient
chr1	842.00	361.81	0.43	53.86	98.34	-0.23
chr2	1,379.00	317.03	0.23	12.44	99.64	-0.02
chr3	1,298.00	359.35	0.28	29.02	99.69	0.97
chr4	945.00	403.08	0.43	82.34	99.58	0.92
chr5	442.00	555.66	1.30	130.98	97.28	0.92
chr6	365.00	369.14	1.00	10.24	97.53	0.97
Total	5,271.00	2,366.07				
Average		394.35	0.612			

### HDGM quality assessment

To assure the quality of genotyping, an integrity evaluation of SNPs was performed among individuals of the mapping population with an average of 95.97% (Supplementary Fig. S4). The SNP marker was anchored to the reference genome of *E. sagittatum* to examine the collinearity between the HDGM and physical location. The results showed that the genetic arrangements of most markers were in the order of the physical map of the *E. sagittatum* genome, with the exceptions of LG1 and 2, which showed a minor departure in the collinearity test (Supplementary Fig. S5). Haplotype mapping was performed for the 109 F1 individuals and the parents using 5,271 SNPs to locate genotyping errors and double recombination. There was no crossover among the six linkage groups, and each individual recombination event was shown to be clear in the haplotype map (Supplementary Fig. S6). The highly purified interspecific F1 population, according to the above results, was appropriate for HDGM development and analysis.

### QTL analysis for flavonoid flavonoids and phenotypes

**Fig. 3:**
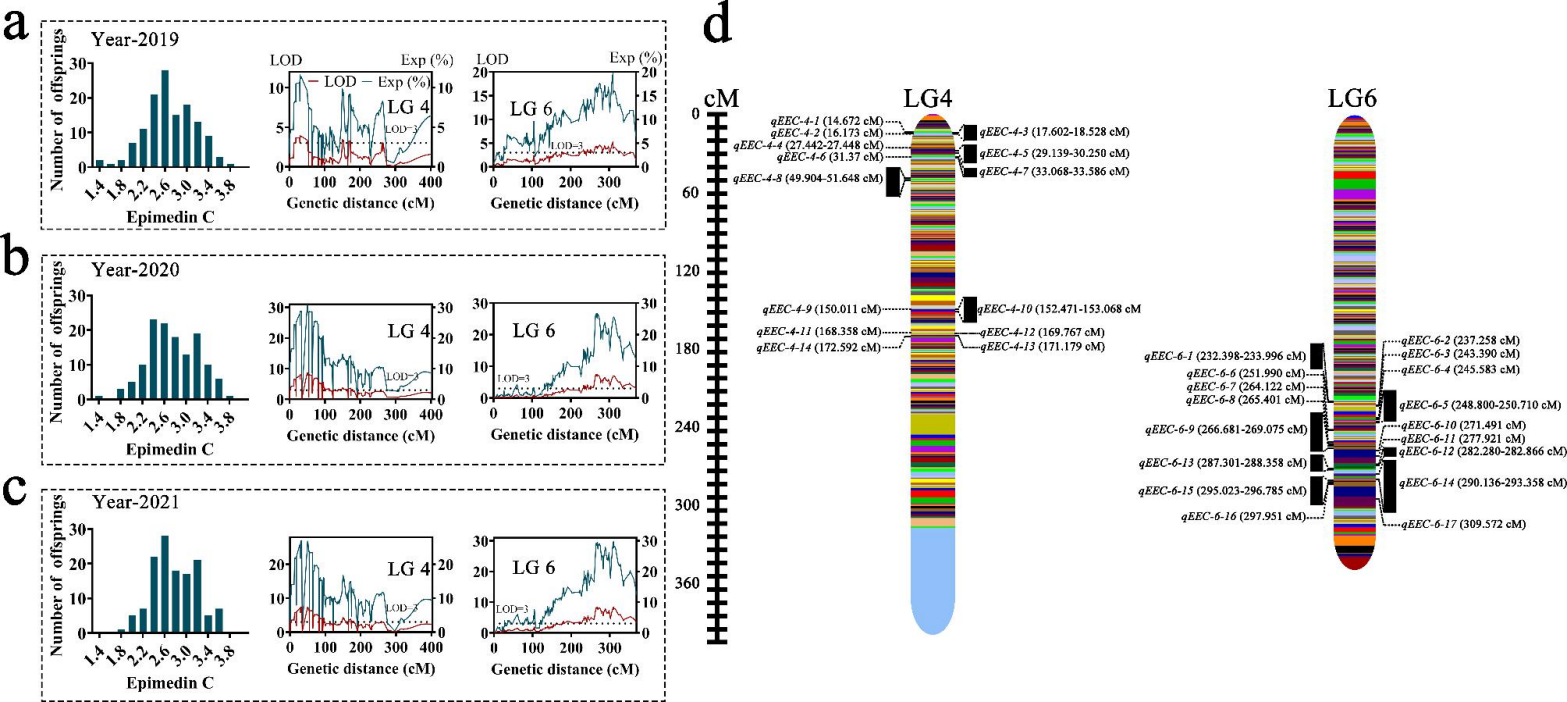
The stable QTL evaluation results for Epimedin C content. Frequency distribution and QTLs for Epimedin C content in 2019 (**a**), 2020 (**b**) and 2021 (**c**). Major stable QTL test results for Epimedin C content over three years (**d**)

**Fig. 4:**
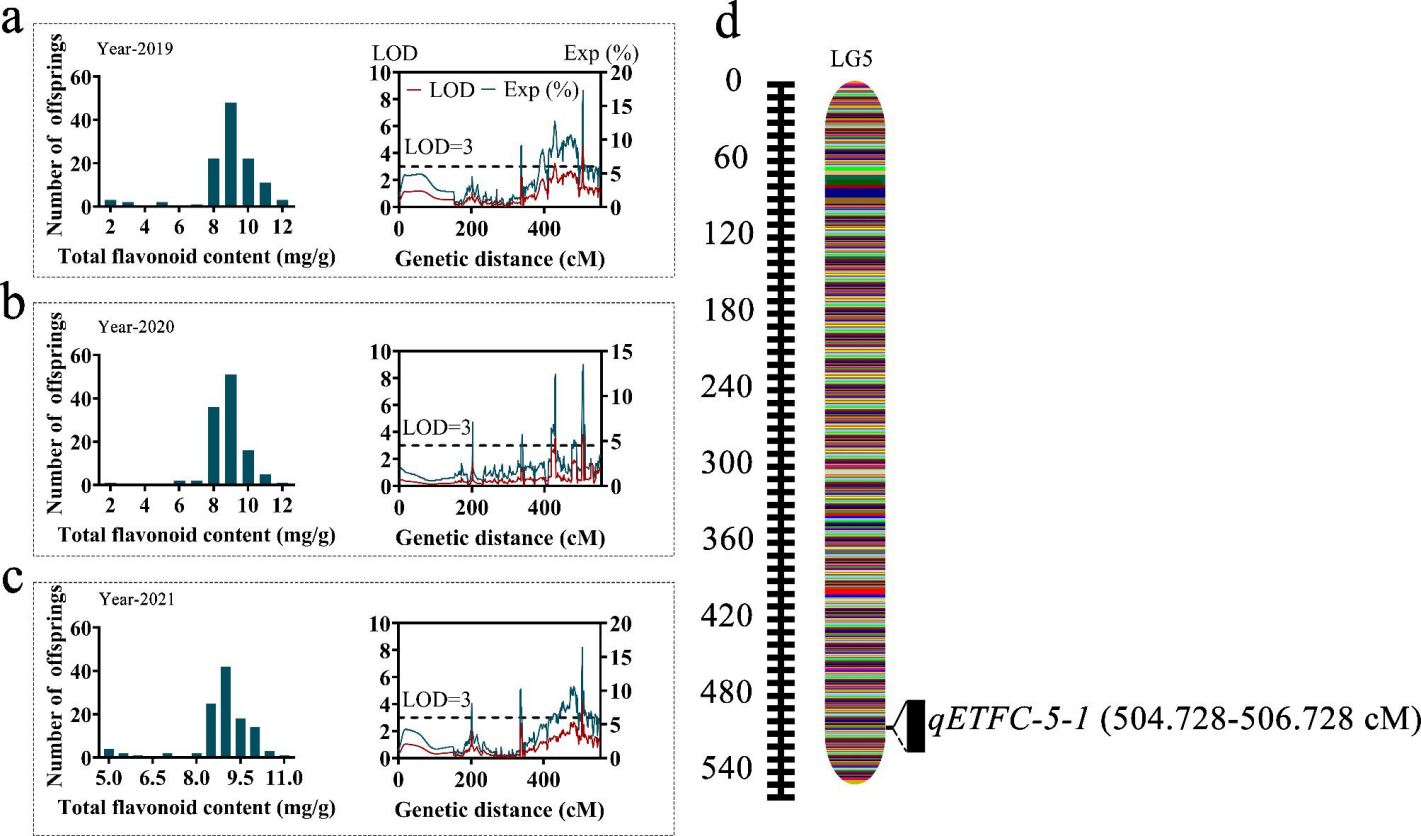
The stable QTLs for the total flavone content. The QTLs and frequency distribution of total flavone content in 2019 (**a**), 2020 (**b**) and 2021 (**c**). The stable QTL assessment result for total flavonoid content within three years (**d**)

Major-effect QTLs covering four traits were analysed separately on the HDGM. For three consecutive years (2019–2021), 31 stable QTLs associated with EC were detected on LG4 and LG6. Of these, 14 QTLs were detected on LG4, which ranged from 14.67 to 172.60 cM in length, with LOD values from 3.00 to 3.86 and explained variance from 4.00 to 11.40%. However, 17 QTLs were positioned on LG6 with a covered distance ranging from 232.40 to 309.57 cM and corresponded to LOD varying from 3.10 to 4.35, explaining variance spanning from 12.30 to 16.80% (Fig. 3 and Supplementary Table S8). For the TFC, only one stable QTL with one associated marker was mapped at 504.73 cM on LG5. The LOD estimate was 3.96, explaining 15.40% of the variance (Fig. 4 and Supplementary Table S8).

**Fig. 5:**
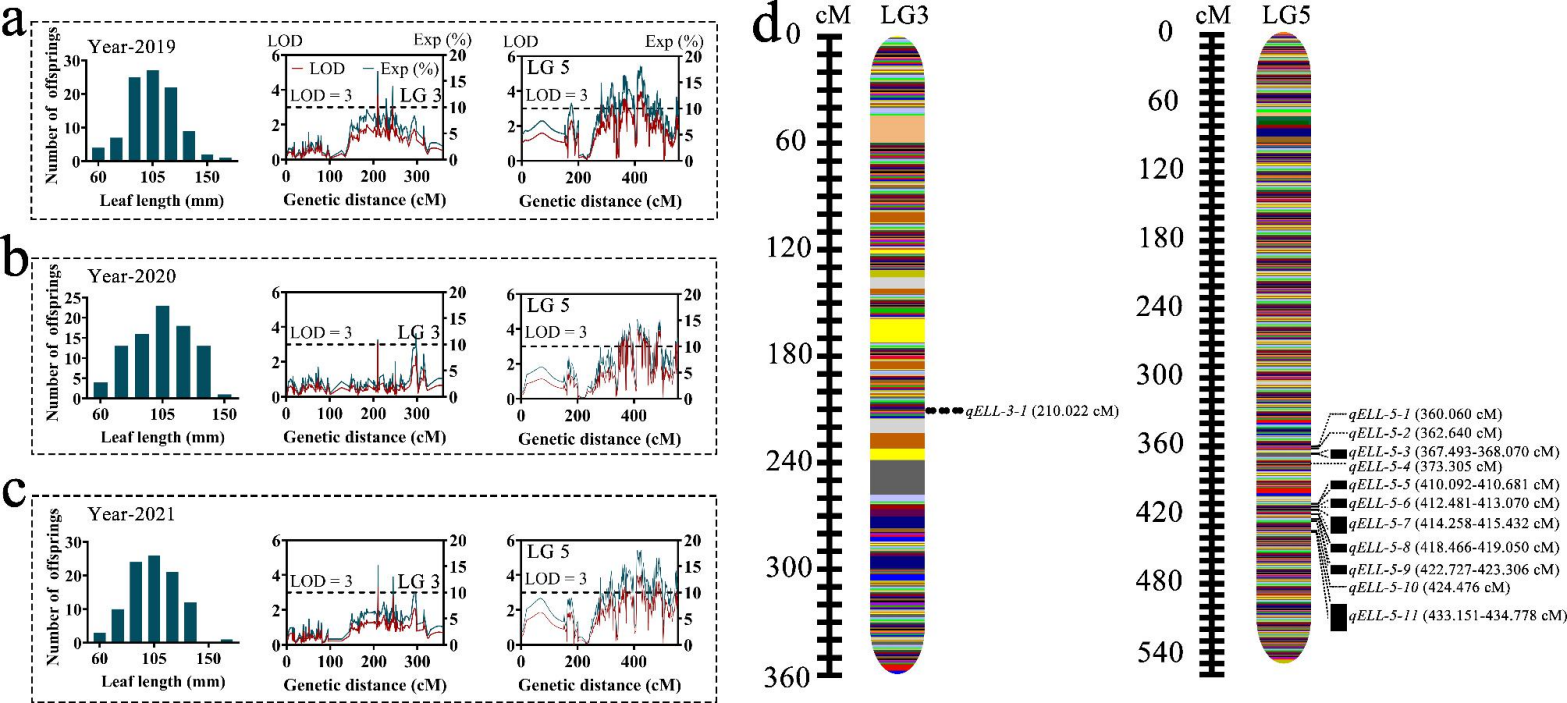
QTLs identified for leaf length in linkage groups three and five. Mapping of QTLs controlling leaf length in 2019 (**a**), 2020 (**b**) and 2021 (**c**). Stable QTL obtained by mapping the leaf length during three consecutive years (**d**)

**Fig. 6:**
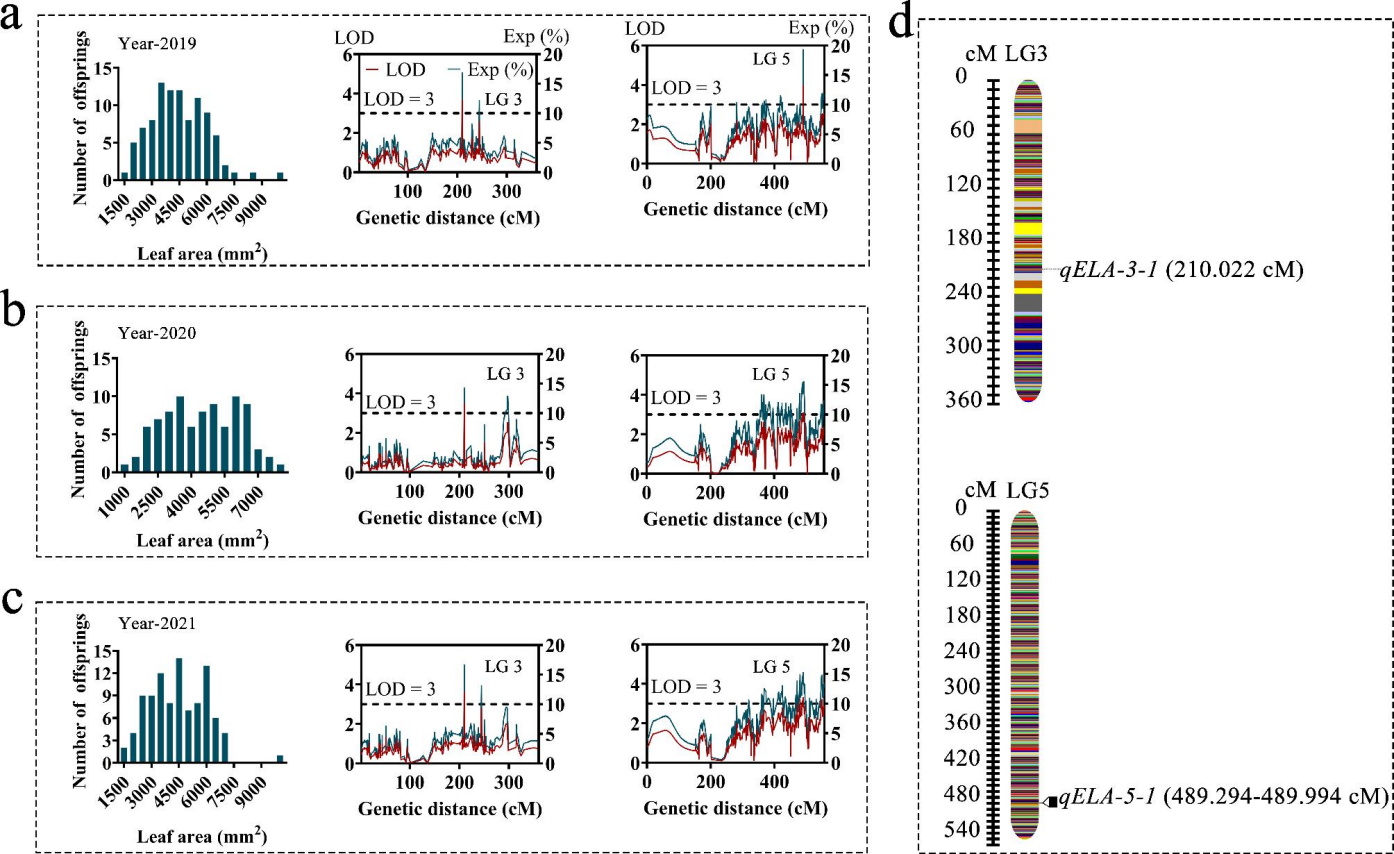
QTL mapping results for the leaf area trait. QTLs for leaf area during 2019 (**a**), 2020 (**b**) and 2021 (**c**) in the F1 mapping population. Location of stable QTLs for leaf area in linkage groups three and five of *Epimedium* (**d**)

QTL analysis identified 12 QTLs targeting LL that were positioned on LG3 and LG5. Among the 12 QTLs, *qELL-3-1* represented a stable QTL throughout the three individual years and was positioned at 210.02 cM onto LG3, with a corresponding LOD value of 3.68 and an explanatory variation of 16.80%. Moreover, 11 stable QTLs of LL were detected on LG5 over the three years (2019–2021), with genetic distances from 360.06 to 434.78 cM and LOD values between 3.26 and 3.80 along with explaining variation ranging from 14.95 to 17.34 (Fig. 5 and Supplementary Table S8). We observed that two LA controlling QTLs spanned LG3 and LG5, of which *qELA-5-1* represented stable QTLs over the three years and covered genetic intervals ranging from 489.29 to 489.99 cM with LOD values of 3.26 and explained a variance of 15.05% (Fig. 6 and Supplementary Table S8). *qELA-3-1* was identified as an important QTL, explaining 16.8% of the variation, and had an LOD value of 3.67. Due to the significant correlation between both LL and LA, QTLs for LL (*qELL-3-1*) and LA (*qELA-3-1*) were discovered in the same regions on LG3 (210.02 cM) (Supplementary Table S8).

## Discussion

### GBS sequencing and the development of markers

HDGM based on molecular marker development is an important foundational study for plants to perform molecular breeding [24]. The distribution and abundance of markers developed from the plant genome influence the regularity and coverage of markers for HDGM [19]. GBS sequencing is an accurate, economical, and efficient method for the acquisition of many SNPs and genotyping in comparison to traditional methods of marker generation [25]. For simplified genome sequencing, it has been suggested that the fragment produced by digestion of the genome with a suitable restriction enzyme can better represent the genome [26]. However, species genome specificity has been neglected in previous studies, and there is usually only one common restriction endonuclease (e.g., *Eco*RI, *Sbf*I, *Mse*I and *Sac*I) to digest genomes of various species [27, 28]. Early studies showed that the application of inappropriate single restriction endonucleases to genome digestion may initiate a maldistribution of identified fragments throughout the plant genome, reducing both the markers generated and the dependability of HDGM [28, 26]. This is consistent with our results that the fragments we harvested for sequencing could better represent the *Epimedium* genome after applying the two restriction endonuclease combinations (*Mse*I and *Sac*I) to genome digestion. This study used the GBS method to generate 46,594 SNPs from the genome, and 14,115 SNP markers were recognized to be considered effective markers for the construction of HDGM for *Epimedium* (Supplementary Table S5). The GC percentage of the sequencing dataset varied from 40.14 to 47.51% (Supplementary Table S4), which was higher than the values obtained from the five *Epimedium* (*E. koreanum, E. pseudowushanense, E. lishihchenii, E. dolichostemon* and *E. acuminatum*) chloroplast genomes (the average percentage of GC was 38.77%)^1^. This might be due to the diverse sources of DNA sequences analysed. Moreover, the GBS sequencing results generated an abundance of genomic information for the species *Epimedium*, which precisely reflects its genomic traits. These results indicate that the GBS approach is a viable method for genotyping and marker generation, which could be adapted in HDGM construction of *Epimedium*.

### Construction of the HDGM of ***Epimedium***

High-density consensus genetic maps are a prerequisite for QTLs to identify tightly linked molecular markers and the location of genes that control quantitative traits and are useful for MAS breeding [19]. The HDGM also provides information about the genome of the assessed population and facilitates recombination landscape analysis within species [21]. Although the consistency of the HDGM is important, there is currently no map available for *Epimedium*. *Epimedium* species have an outbreeding system and lack an internal barrier to hybridization (high incompatibility and crossover ability) [29], and perennial plants are more complicated to construct HDGM than homozygous annual herbaceous plants due to their lengthy generation spacing and high heterozygosity [30, 31]. In this study, an F1 family generated from a hybrid between *E. leptorrhizum* and *E. sagittatum* was employed to develop *Epimedium* HDGM. We used the GBS protocol to generate abundant SNPs and utilized the *Epimedium* genome to call SNPs. The number of molecular markers in the HDGM is one of the important indicators to assess its quality [24]. Since the construction of the first genetic map, most genetic maps have been based on traditional markers such as simple sequence repeats (SSRs), the number of available markers is small, and their mapping resolution accuracy is limited [21]. A total of 14,115 SNP markers were detected, of which 5,271 were mapped on the first consensus HDGM of *Epimedium*, and the mean distance between markers was 0.61 cM (Fig. 2; Table 1). Compared with the linkage map constructed based on traditional markers (e.g., SSR), the *Epimedium* consensus HDGM added more than 3,000 markers, and the mean marker distance was reduced by 10.21 cM (the shortest average distance between traditional markers is 10.82 cM) [19]. Haplotype maps, the integrity of mapping individuals on the HDGM, and collinearity were used to assess HDGM reliability [14, 32]. The haplotype maps and the integrity of the individuals on the HDGM and the collinearity revealed that the high-density SNPs were accurately arranged within LGs (Supplementary Figs. S4-S6), which indicated the consistency of the HDGM. The consensus HDGM is the first *Epimedium* high-accuracy map with a large number of SNP markers, suitable distance, high resolution, and balanced various *Epimedium* HDGM indices, which can be used to identify QTLs for important *Epimedium* traits.

### QTL mapping

The F1 cross-pollinated (CP) model differs from the backcross, recombinant inbred lines, and double haploid populations used for mapping. The MapQTL program with interval mapping (IM) is appropriate technology for performing QTL evaluation in the CP model [14], and it has been effectively used in various species, such as pepper [32] and goji berry [14]. QTL mapping analysis for secondary metabolite and leaf size-related traits using HDGM is an effective prediction method for quantitative trait mapping [14]. Although specific secondary metabolites have important medicinal and commercial value, few QTLs related to modifying secondary metabolite content in *Epimedium* have been identified. Epimedin C is a glycosylated kaempferol derivative, and it is known that most GT proteins are related to glycosylation in plant secondary metabolism [33, 34]. Using QTL analysis, we evaluated two *Epimedium* secondary metabolite traits and discovered that EC had a significant correlation with its genetic variation. The identification of 31 stable QTLs associated with EC varied in both their proportion of phenotypic variance explained and significance, indicating that the content of EC in *Epimedium* is affected by quantitative genetics (Fig. 3 and Supplementary Table S8). These results are in line with genetic analyses of metabolites in eucalyptus [35] and hops [8]. However, only one stable QTL for the TFC trait on LG5 was discovered (Fig. 4 and Supplementary Table S8). The identification of only one TFC related QTL site might be due to TFC being composed of multiple compounds or to slight variations in the TFC among the mapping populations.

Leaf size affects plant biomass [9] and is also an essential economic trait of *Epimedium*, and the leaves are the major organs used as *Epimedium* medicinal herbs [4]. QTLs have been successfully used to discover candidate genes for plant leaf traits, including those of goji berry [14], grape [36] and wolfberry [37]. In the three years, 12 stable QTLs for LL were discovered, with LG3 and LG5 regarded as hotspot locations for LL-related traits (Fig. 5 and Supplementary Table S8). In addition, we observed one LA related QTL in LG3 and LG5 (Fig. 6 and Supplementary Table S8). A significant correlation was found between LL and LA, and it is interesting that we also observed a colocalization phenomenon in LG3, although there was only one marker for this locus. Additionally, QTLs corresponding to LA and LL shared common positions on LG3 and LG5 throughout the three years. The colocalization of traits associated with QTLs usually reflects dual genomic regions in addition to positive significant correlations among different traits [14]. Colocalization among different traits has also been found in other species, such as the fruit diameter and pericarp thickness of peppers, which have three common locations on the genetic map [38]. In addition, LL, LA, fruit length, and leaf diameter of goji berries have also been observed to colocalize [14], indicating that the colocalization phenomenon of QTLs for various traits discovered in the common genomic area may be due to linkage or pleiotropy [38, 14]. In *Epimedium*, these markers might be used for leaf size and flavonoid content related trait breeding.

## Materials and methods

### Mapping population sampling

To construct an HDGM, *E. leptorrhizum* (female) and *E. sagittatum* (male) with significant differences in flavonoid content and leaf size were used as the hybrid parents. *E. sagittatum*, flowers white or yellow, ca. 8 mm or less in diam; yellow petals; leaflets ovate to ovate-lanceolate, 5–19 × 3–8 cm. *E. leptorrhizum*, flowers white, tinged with rose or deep rose, large, 3.5-4 cm in diameter; leaflets narrowly ovate or ovate, 3–10 × 2–5 cm (Fig. 1). The TFC of the male parent was higher than that of the female parent, and the content of EC was lower (Fig. 1). The hybrid parents were discovered in the field and cultivated in Wuhan Botanical Garden. In February-April 2017, a cross between *E. leptorrhizum* and *E. sagittatum* produced 109 hybrid seedlings. Hybrid seedlings were cultivated in a common garden with the hybrid parents for two years. To determine the purity of the offspring, four pairs of EST-SSR primers (Supplementary Fig. S7a) with amplified homozygous bands in the parents and hybrid progenies were used. Genotyping results showed that all 109 progenies were true hybrids and were used for subsequent analysis (Supplementary Fig. S7b). Every year from 2019 to 2021, the leaf tissues of the 109 F1 offspring plants and the two parents were sampled at the flowering stage and then frozen and stored at -80 °C for future evaluation.

### Extraction of flavonoids and analysis

The leaves of the 109 F1 hybrid seedlings and the two parents were dried in the shade. The dried leaves were ground into powder in a mortar with liquid N_2_ and then filtered with a 50-mesh sieve. Five milliliters of EtOH-H_2_O (70:30, v/v) was used for every 0.05 g of powder, and then the solution was filtered with a 0.22-micron membrane. The EC was analysed by HPLC using a previously described method [4]. All analyses were repeated three times using the identical HPLC system.

The TFC was examined according to the colorimetric method [39]. In a microtiter tube, six milliliters of pure water were added to one milliliter of sample solution or standard catechin solution. After adding 300 µl of 5% NaNO_2_, the mixture was incubated for five minutes. Afterwards, 450 µl of 10% AlCl_3_ was added, and the mixture was allowed to stand for five minutes before adding three milliliters of one mole of NaOH and reacting for 30 min at room temperature. A spectrophotometer (Bio Tek Instruments, Winooski, VT, USA) was then used to measure the absorbance at 510 nanometers. The TFC was determined in milligrams of catechin equivalent (CAE) per gram of dried *Epimedium* powder (mg CAE/g).

### Phenotypic data analysis

From 2019 to 2021, the LL and LA of the 109 F1 hybrid seedlings and two parents were measured from fresh leaves during the annual flowering period (mid-April) using LA-S software (Hangzhou Wanshen Detection Technology Co., Ltd.). All measurements were performed five times to guarantee the reliability of the results.

### DNA extraction, library construction and sequencing

Using a modified cetyltrimethylammonium bromide (CTAB) method [40], DNA was extracted, and the DNA produced was evaluated using agarose gel electrophoresis. Our GBS libraries of the 111 genomic DNA samples were constructed using the previously described protocol [41]. *Mse*I and *Sac*I were used to digest 200 nanograms of DNA from each sample for an hour at 37 °C. After adapter ligation, the fragments were amplified with KOD-Plus-Neo (TOYOBO., LTD., Life Science Department OSAKA JAPAN) using the two sequencing primers. Thereafter, the fragments were separated using a 2% agarose cartridge to capture a narrow distribution range of 380–480 bp and sequenced using 150 bp paired-end reads on the Illumina HiSeqTM 2500 platform (Illumina, Inc. ; San Diego, CA, US).

### Genotyping and SNP identification

For quality control, the sequencing data were analysed using FASTP (V. 0.20.1) tool [42]. Poly-G and poly-X read tails and reads with lengths < 15 bp were discarded. Using the BWA software package (v.0.7.17) [43], the filtered clean reads were aligned to the *E. sagittatum* genome. Then, the screened SNPs were divided into six LGs by mapping the locations on the *E. sagittatum* genome. Finally, mapping was conducted using read pairs with a single unique location in the *E. sagittatum* genomes. Haplotype Caller in GATK (v.4.1.9.0) [44] was used to find SNPs and inserts, and the variants were filtered. The remaining SNPs were then coded based on the division patterns ef×eg, hk×hk and lm×ll.

### HDGM construction and evaluation

JoinMap 5 [45] was used for HDGM construction under cross-pollinator (CP) patterns. The HDGM was developed using the regression mapping technique (Rec = 0.40, LOD > 1.0, Jump = 5, and ripple = 1). The markers of each LG were sorted using Kosambi’s mapping function, and HDGM distances were calculated in centimorgans (cM). Collinearity analysis and a haplotype map were used to assess the quality of the HDGM [46, 47]. The haplotype map [47] and collinearity analysis methods [46] were evaluated according to a previously described protocol.

### QTL mapping

QTL analyses were conducted on three consecutive years for two leaf size (LL and LA) traits and two flavonoid content (EC and TFC) related traits using MapQTL®v6.0 software [48], which employed the interval mapping (IM) method to evaluate QTL loci among 6 LGs for the HDGM. The LOD score significance threshold for each trait was assessed using a permutation test, and the IM model was used to search for potential QTLs on each LG. A QTL detected in all three years was defined as a stable QTL [15].

### Statistical analysis

The analysis of variance, Pearson correlation, and frequency distribution were conducted using SPSS v. 25 (SPSS Inc., Chicago, IL, United States). The significance level was established at p < 0.05 for a two-tailed correlation test. The delineation result graph was generated using GraphPad Prism 9.0.0 software (GraphPad Software, Inc.).

## Electronic supplementary material

Below is the link to the electronic supplementary material.

Supplementary Material 1

## Data Availability

The datasets generated or analysed during this study are included in this article (and its Additional file) or are available from the corresponding author on reasonable request. All raw data were submitted to the NCBI Sequence Read Archive: BioProject ID PRJNA910928.

## Abbreviations

ANOVA: Analysis of variance
CAE: Catechin equivalent
CP: Cross-pollinated
CTAB: Cetyltrimethylammonium bromide
CV%: Coefficient of variation
EC: Epimedin C
EST-SSR: Expressed sequence tags- simple sequence repeat
GBS: Genotyping by sequencing
GC: Guanine-cytosine
HDGM: High-density genetic map
HPLC: High-performance liquid chromatography
IM: Interval mapping.
LA: Leaf area
LG: Linkage groups
LL: Leaf length
LOD: Logarithm of odds
MAS: Marker-assisted selection
QTL: Quantitative trait loci
SNP: Single nucleotide polymorphism
TFC: Total flavonoid content
