# Supplementary material for: Construction of the first high-density genetic linkage map and QTL mapping of flavonoid and leaf-size related traits in Epimedium
Source: BMC Plant Biol. 2023 May 25;23:278. doi: 10.1186/s12870-023-04257-0 (PMC10210407; doi:10.1186/s12870-023-04257-0)
Supplement: Supplementary file 1 — Supplementary Material 1 [file 12870_2023_4257_MOESM1_ESM.docx]

## Supplementary figures and tables


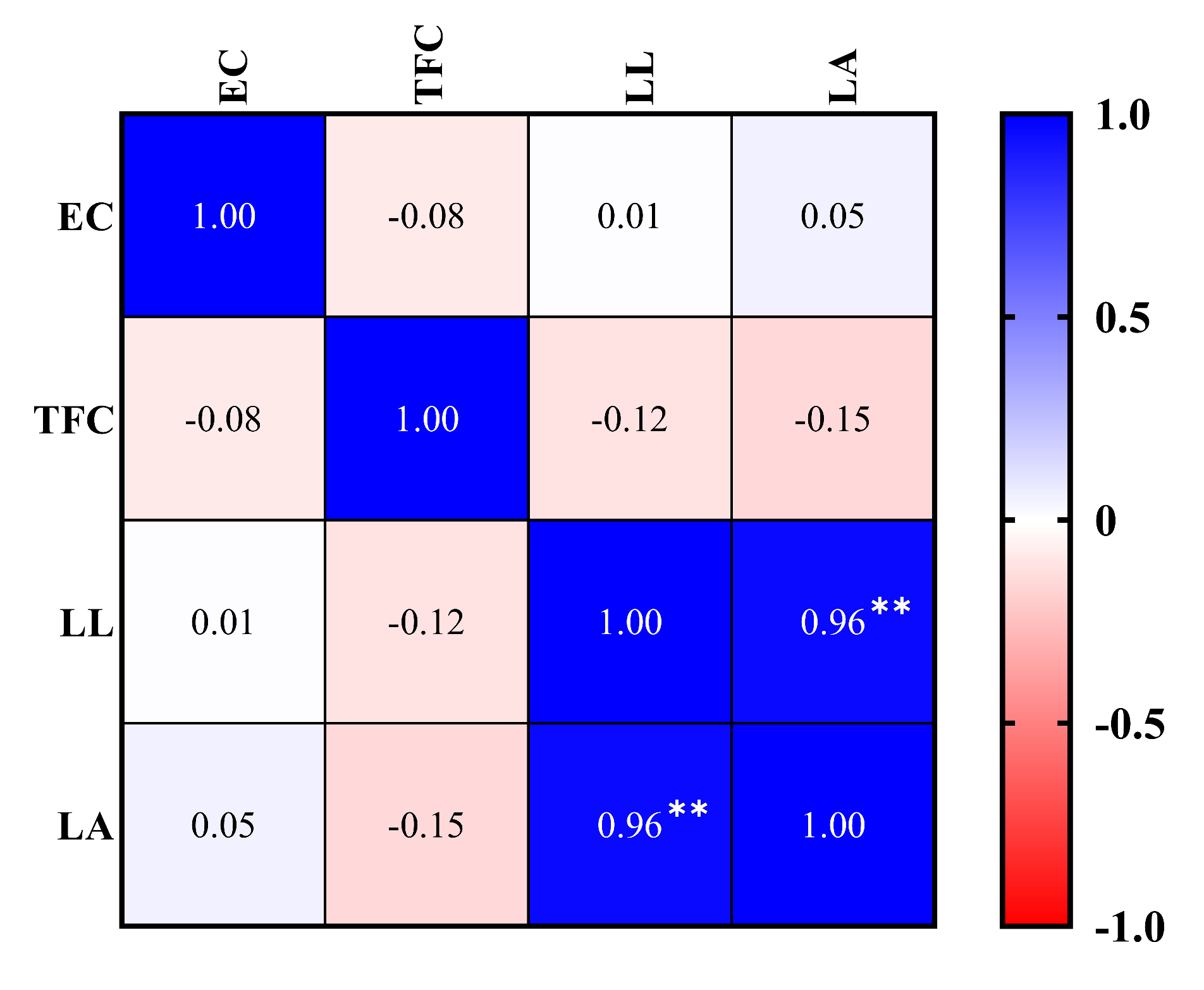


#### Fig S1 Correlation analysis between traits for QTL analysis.

* Correlation is significant at *p*=0.05 (2-tailed)


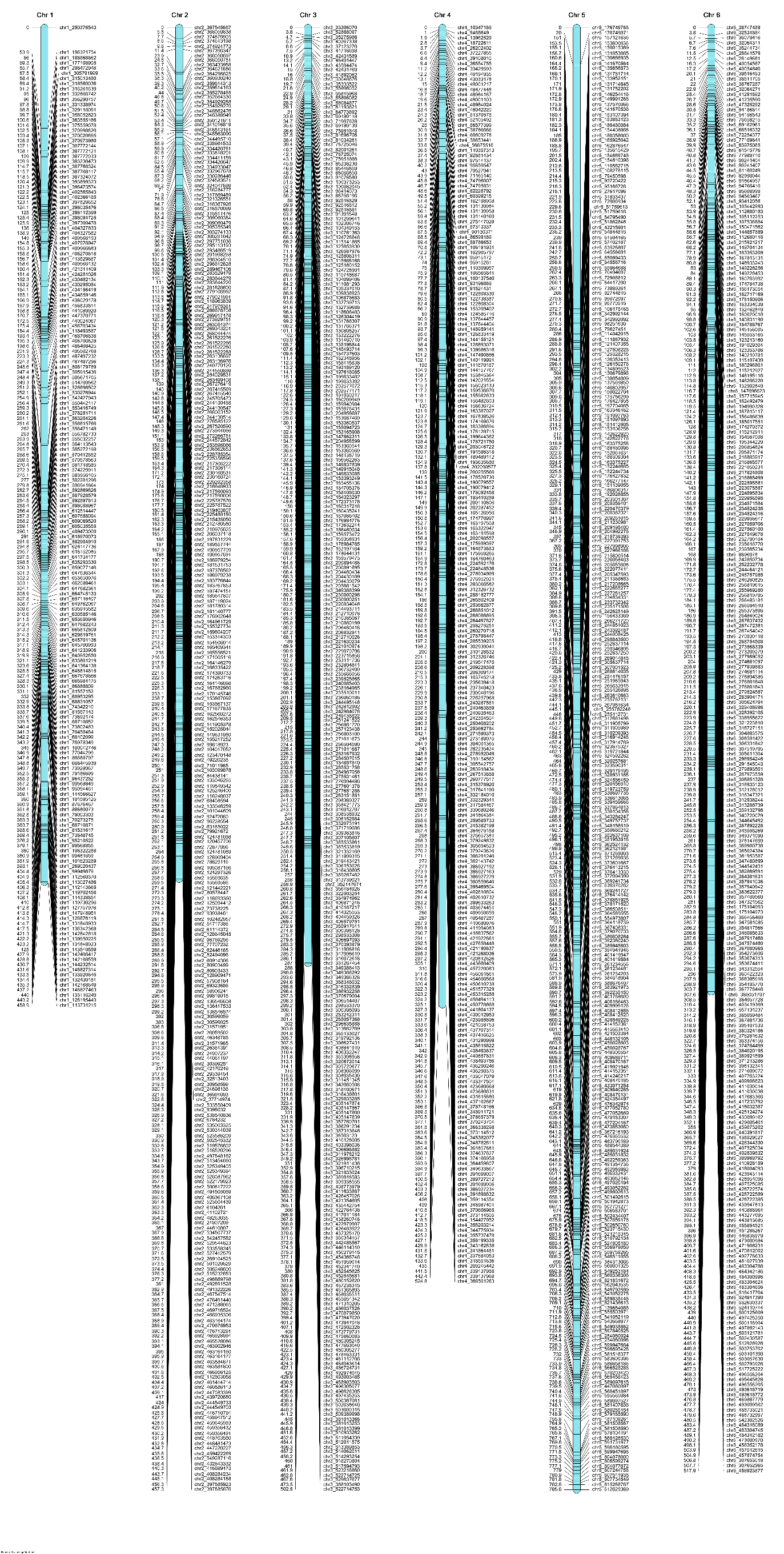


#### Fig S2 The paternal high-density genetic map.


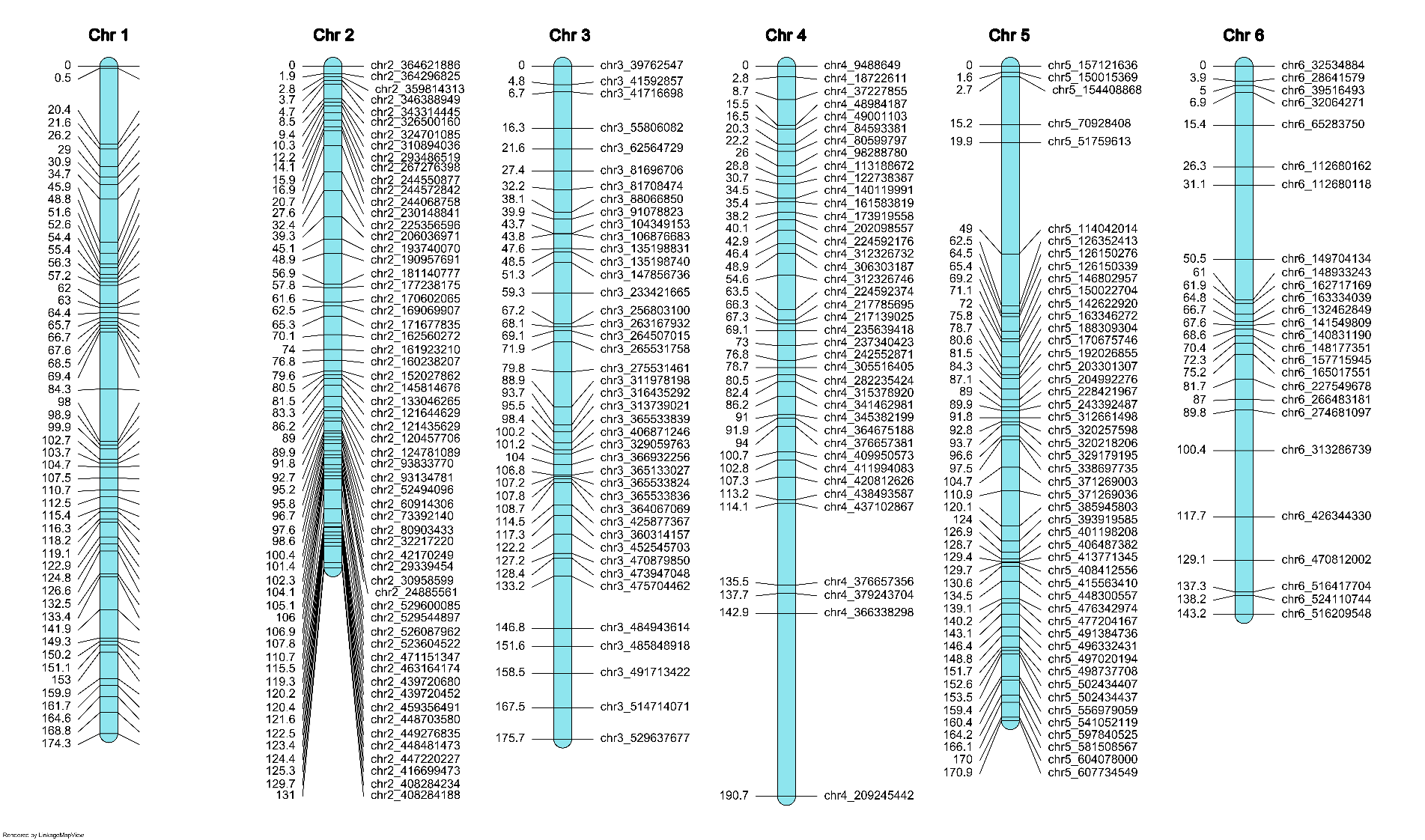


#### Fig S3 The maternal high-density genetic map.


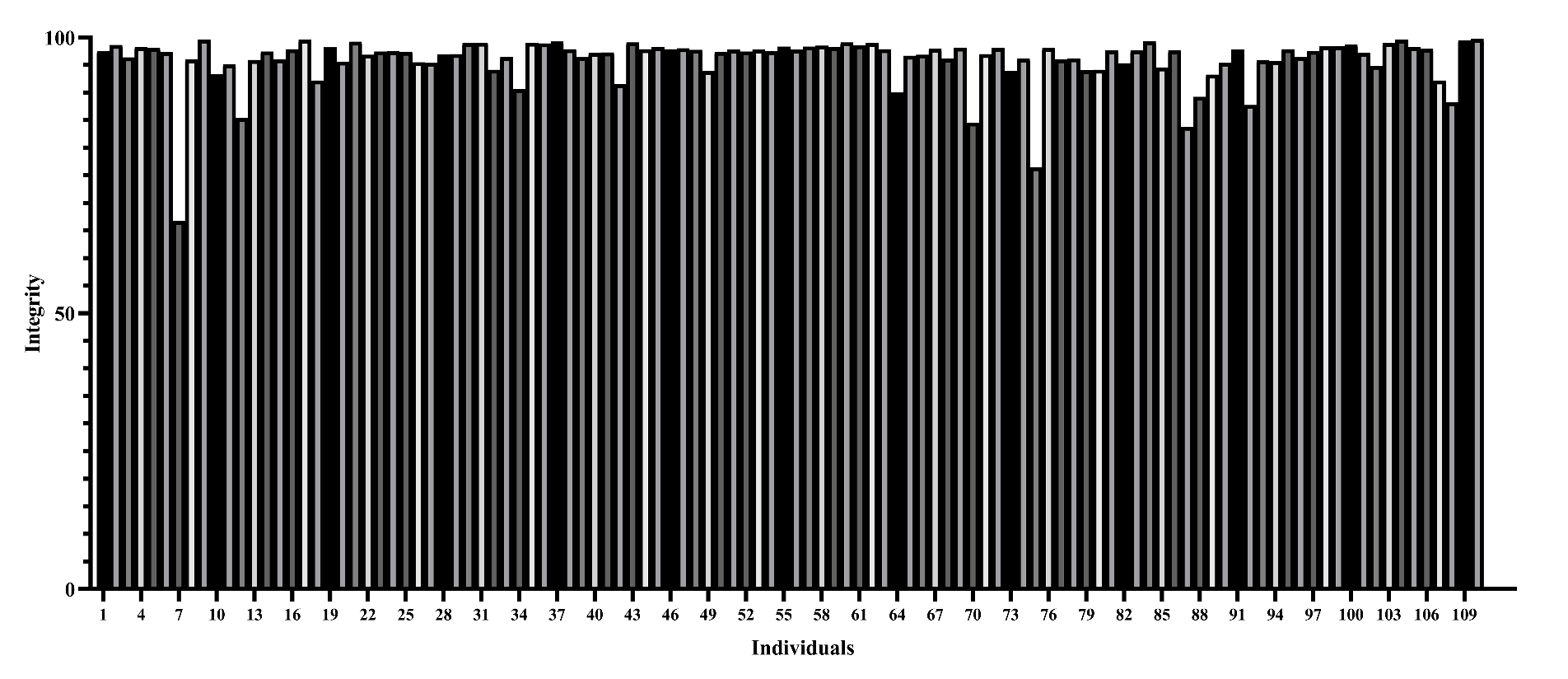


#### Fig S4 The integrity distribution map of all individuals.


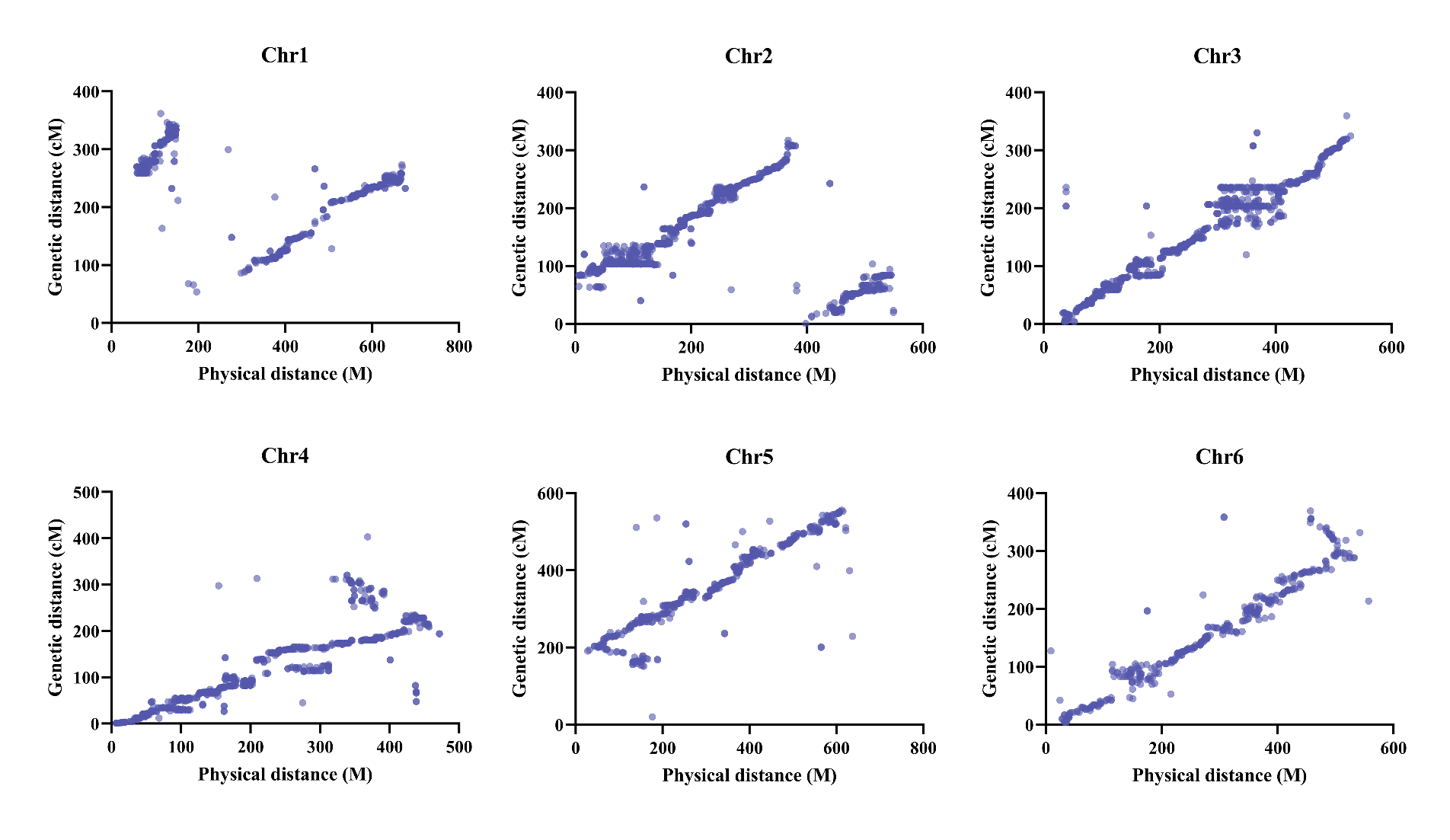


#### Fig S5 Collinearity analysis between the consensus map and the genome of *Epimedium sagittatum*.


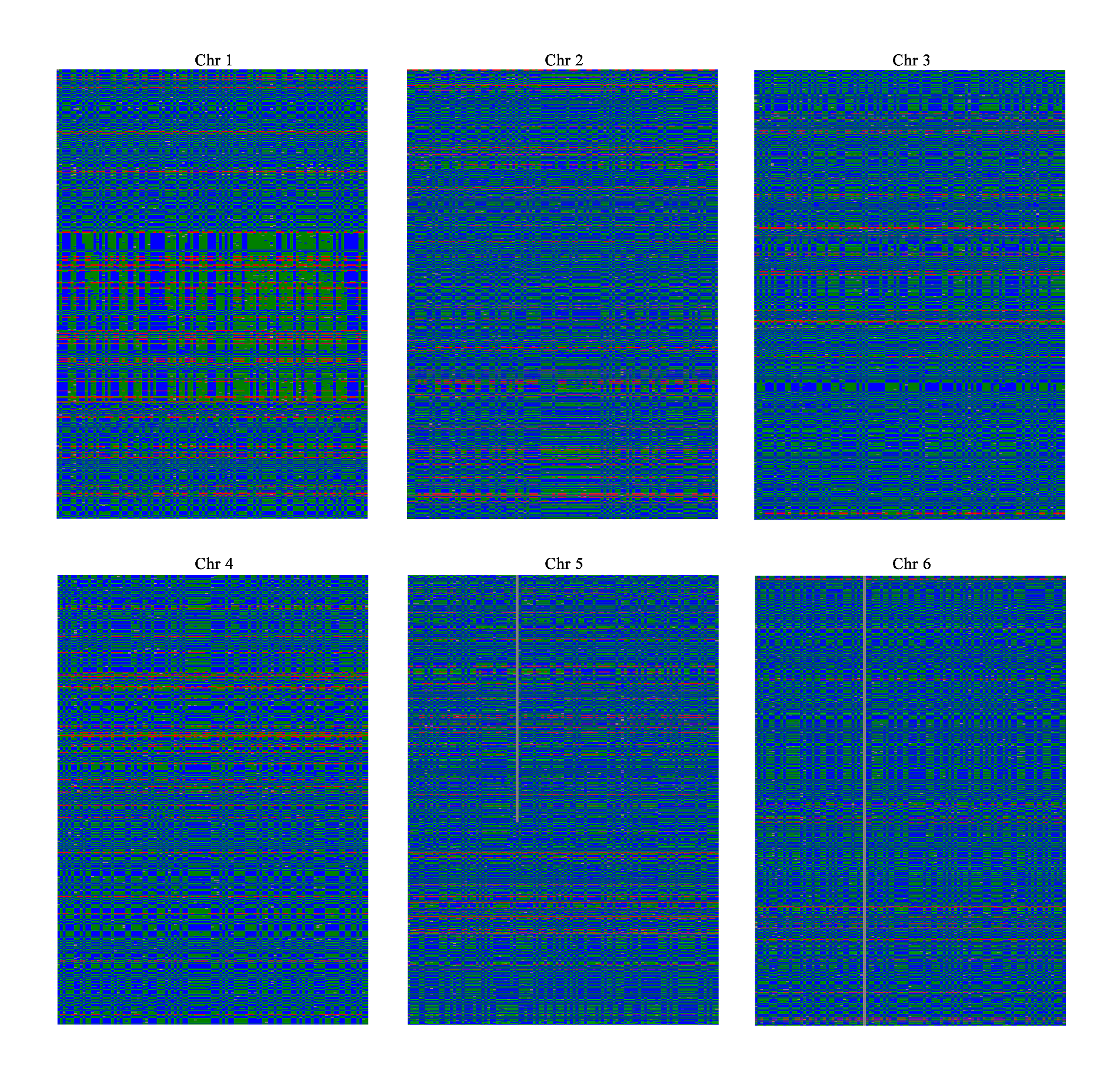


#### Fig S6 Recombination bin map of 109 F1 population. Green: *Epimedium sagittatum*; blue: *Epimedium leptorrhizum*; red: heterozygote; gray: missing data.


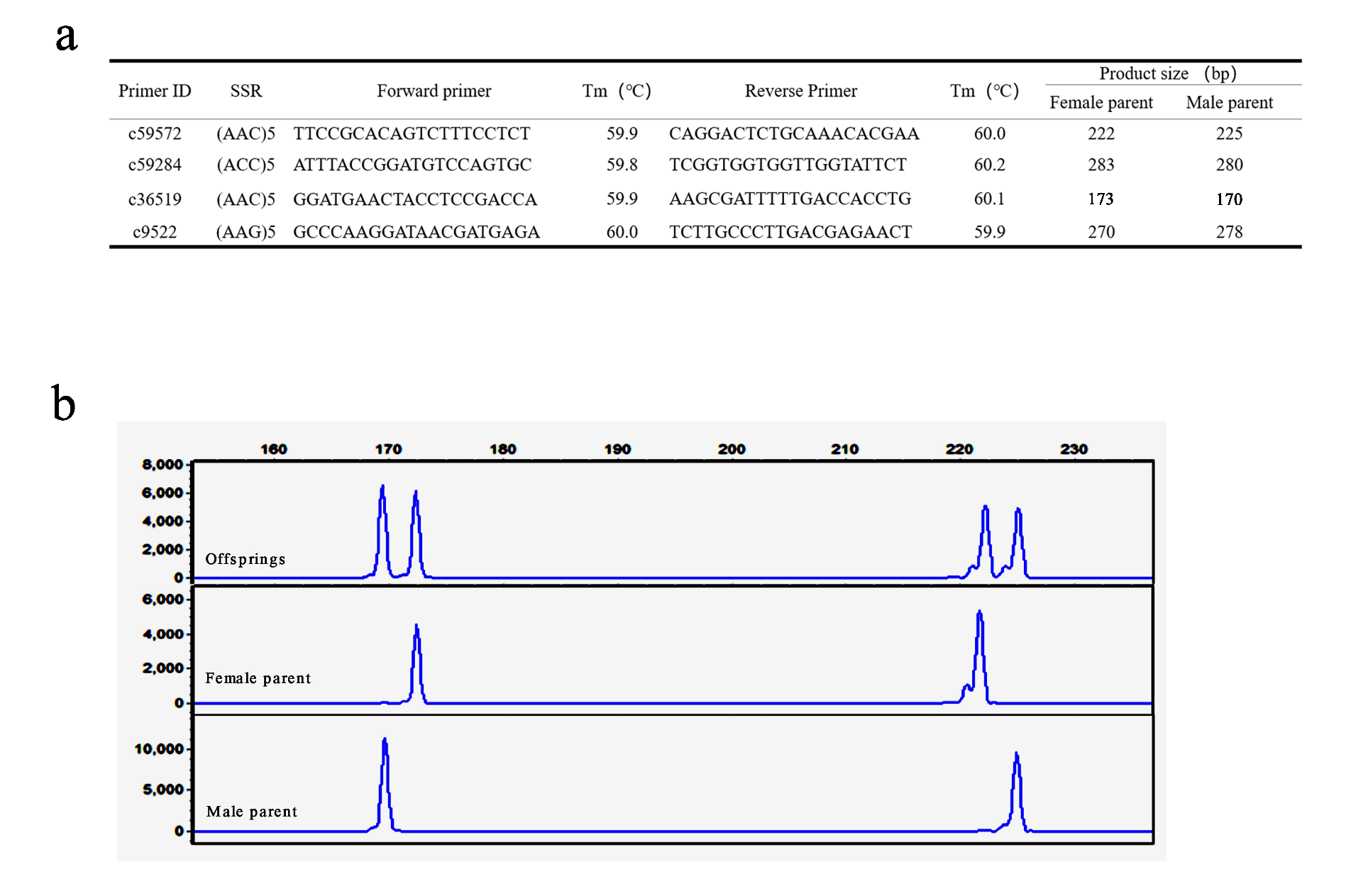


#### Fig S7 Primers and results of hybrid progeny genotyping. (a) Detailed information of EST-SSR primers. (b) Genotyping results based on EST-SSR primers.

#### Table S1 Comprehensive analysis of variance (ANOVA) between 3 individual years (2019-2021) of trait data collected from the 109 offspring of F1.

| Source | | Sum of Squares | df | Mean Square | F | P |
| --- | --- | --- | --- | --- | --- | --- |
|  | Years | 0.5291 | 2 | 0.2645 | 1.337 | 0.2637 |
| EC | Offspring | 77.14 | 390 | 0.1978 |  |  |
|  | Total | 77.66 | 392 |  |  |  |
|  | Years | 0.9647 | 2 | 0.4823 | 0.2648 | 0.7675 |
| TFC | Offspring | 606.5 | 333 | 1.821 |  |  |
|  | Total | 607.4 | 335 |  |  |  |
|  | Years | 158.6 | 2 | 79.30 | 0.2096 | 0.8111 |
| LL | Offspring | 98761 | 261 | 378.4 |  |  |
|  | Total | 98919 | 263 |  |  |  |
|  | Years | 92546 | 2 | 46273 | 0.0207 | 0.9795 |
| LA | Offspring | 582712943 | 261 | 2232617 |  |  |
|  | Total | 582805489 | 263 |  |  |  |

EC, Epimedin C; TFC, total flavonoid content; LL, leaf length; LA, leaf area

#### Table S2. Descriptive statistics among the data for the four traits based on the three individual years (2019-2021).

| Traits | Years | Mean | Standard Deviation | Standard error | Maximum | Minimum | Range | Skewness | Kurtosis | variance | coefficient of variation % |
| --- | --- | --- | --- | --- | --- | --- | --- | --- | --- | --- | --- |
|  | 2019 | 2.687 | 0.462 | 0.040 | 3.808 | 1.378 | 2.430 | -0.257 | 0.145 | 0.214 | 17.195 |
| EC | 2020 | 2.758 | 0.464 | 0.041 | 3.755 | 1.356 | 2.398 | -0.049 | -0.365 | 0.215 | 16.827 |
|  | 2021 | 2.771 | 0.406 | 0.035 | 3.622 | 1.869 | 1.754 | 0.045 | -0.619 | 0.164 | 14.636 |
|  | 2019 | 8.918 | 1.758 | 0.165 | 11.737 | 1.859 | 9.878 | -2.206 | 6.780 | 3.091 | 19.712 |
| TFC | 2020 | 8.789 | 1.076 | 0.101 | 11.543 | 1.594 | 9.949 | -2.496 | 17.379 | 1.159 | 12.247 |
|  | 2021 | 8.854 | 1.108 | 0.104 | 11.192 | 4.964 | 6.227 | -1.839 | 4.575 | 1.227 | 12.513 |
|  | 2019 | 105.280 | 18.827 | 2.007 | 145.871 | 61.057 | 84.814 | -0.025 | -0.716 | 354.473 | 17.883 |
| LL | 2020 | 103.381 | 21.056 | 2.245 | 144.910 | 59.369 | 85.540 | -0.213 | -0.833 | 443.340 | 20.367 |
|  | 2021 | 104.356 | 18.368 | 1.958 | 138.481 | 64.712 | 73.768 | -0.103 | -0.889 | 337.366 | 17.601 |
|  | 2019 | 4481.747 | 1431.719 | 151.762 | 8366.822 | 1777.667 | 6589.155 | 0.209 | -0.402 | 2049819.081 | 31.946 |
| LA | 2020 | 4436.442 | 1669.051 | 176.919 | 8081.898 | 1217.431 | 6864.467 | 0.044 | -0.919 | 2785731.091 | 37.621 |
|  | 2021 | 4460.552 | 1411.170 | 149.584 | 7038.900 | 1508.180 | 5530.720 | -0.039 | -0.937 | 1991399.551 | 31.637 |

EC, Epimedin C; TFC, total flavonoid content; LL, leaf length; LA, leaf area

#### Table S3 Test for normal distribution of the four traits data collected from the 109 offspring of F1.

| Test for normal distribution | Epimedin C (EC) | | | Total flavonoid content (TFC) | | | Leaf length (LL) | | | Leaf area (LA) | | |
| --- | --- | --- | --- | --- | --- | --- | --- | --- | --- | --- | --- | --- |
|  | 2019 | 2020 | 2021 | 2019 | 2020 | 2021 | 2019 | 2020 | 2021 | 2019 | 2020 | 2021 |
| Anderson-Darling test |  |  |  |  |  |  |  |  |  |  |  |  |
| A2* | 0.3146 | 0.557 | 0.7266 | 7.539 | 3.537 | 6.944 | 0.4664 | 0.5694 | 0.4215 | 0.4045 | 0.4984 | 0.4285 |
| P value | 0.5403 | 0.1477 | 0.0568 | <0.0001 | <0.0001 | <0.0001 | 0.2469 | 0.136 | 0.3169 | 0.3477 | 0.2055 | 0.3049 |
| Passed normality test (alpha=0.05) | Yes | Yes | Yes | No | No | No | Yes | Yes | Yes | Yes | Yes | Yes |
| P value summary | ns | ns | ns | **** | **** | **** | ns | ns | ns | ns | ns | ns |
| Shapiro-Wilk test |  |  |  |  |  |  |  |  |  |  |  |  |
| W | 0.9909 | 0.9869 | 0.9816 | 0.762 | 0.8038 | 0.8028 | 0.9863 | 0.9755 | 0.9832 | 0.9764 | 0.9796 | 0.9758 |
| P value | 0.5569 | 0.2432 | 0.0723 | <0.0001 | <0.0001 | <0.0001 | 0.4166 | 0.0942 | 0.2527 | 0.0776 | 0.1813 | 0.0694 |
| Passed normality test (alpha=0.05) | Yes | Yes | Yes | No | No | No | Yes | Yes | Yes | Yes | Yes | Yes |
| P value summary | ns | ns | ns | **** | **** | **** | ns | ns | ns | ns | ns | ns |
| Kolmogorov-Smirnov test |  |  |  |  |  |  |  |  |  |  |  |  |
| KS distance | 0.05475 | 0.06374 | 0.07221 | 0.2345 | 0.139 | 0.2094 | 0.06152 | 0.06799 | 0.0696 | 0.07393 | 0.07223 | 0.06005 |
| P value | >0.1000 | >0.1000 | 0.0906 | <0.0001 | <0.0001 | <0.0001 | >0.1000 | >0.1000 | >0.1000 | >0.1000 | >0.1000 | >0.1000 |
| Passed normality test (alpha=0.05) | Yes | Yes | Yes | No | No | No | Yes | Yes | Yes | Yes | Yes | Yes |
| P value summary | ns | ns | ns | **** | **** | **** | ns | ns | ns | ns | ns | ns |

#### Table S4 Quality evaluation of GBS data for parents and progenies

| Sample | | Clean bases | Clean reads | Q20 (%) | Q30 (%) | GC content (%) | Mapping rate (%) | Depth | Coverage (%) |
| --- | --- | --- | --- | --- | --- | --- | --- | --- | --- |
| Parents | Male | 13,282,628,792 | 106,416,658 | 98.49 | 94.75 | 40.65 | 99.59 | 3.19 | 15.44 |
|  | Female | 12,404,524,854 | 100,046,576 | 98.52 | 94.84 | 40.14 | 99.76 | 3.01 | 13.87 |
| Progeny | Average | 1,479,145,143 | 10,989,287 | 98.51 | 94.77 | 41.15 | 95.53 | 0.36 | 5.38 |
|  | Maximum | 4,461,746,689 | 33,063,778 | 98.63 | 95.11 | 47.51 | 99.56 | 1.07 | 9.60 |
|  | Minimum | 793,946,183 | 6,179,886 | 98.29 | 94.15 | 40.20 | 66.2 | 0. 34 | 2.62 |
|  | Total | 161,226,820,592 | 1,197,832,240 |  |  |  |  |  |  |
| All sample |  | 186,913,974,238 | 1,404,295,474 |  |  |  |  |  |  |

#### Table S5 Statistical analysis of the genetic map marker types.

| Chromosome | SNPs obtained after filtering | Segregating pattern (SNPs used for mapping) | | | |
| --- | --- | --- | --- | --- | --- |
|  |  | lm×ll | hk×hk | ef×eg | Total |
| chr1 | 8807 | 2067 | 73 | 13 | 2153 |
| chr2 | 8942 | 2526 | 71 | 11 | 2608 |
| chr3 | 6581 | 2160 | 40 | 6 | 2206 |
| chr4 | 6409 | 1984 | 33 | 9 | 2026 |
| chr5 | 8797 | 2619 | 64 | 10 | 2693 |
| chr6 | 7058 | 2393 | 29 | 7 | 2429 |
| Total | 46,594 | 13,749 | 310 | 56 | 14,115 |

#### Table S6 Description of the basic characteristics of the six linkage groups in males.

| Linkage/n Group ID | Total/n Marker | Total/n Distance (cM) | Average/n Distance (cM) | Max/n Gap (cM) | Gap/n<5 cM (%) | Spearman/n correlation coefficient |
| --- | --- | --- | --- | --- | --- | --- |
| chr1_P1 | 842 | 458.85 | 0.55 | 53.86 | 98.34 | -0.23 |
| chr2_P1 | 1379 | 457.27 | 0.33 | 12.434 | 98.98 | 0.02 |
| chr3_P1 | 1298 | 502.48 | 0.39 | 29.024 | 99.46 | 0.97 |
| chr4_P1 | 943 | 524.75 | 0.56 | 82.341 | 99.15 | 0.92 |
| chr5_P1 | 442 | 785.62 | 1.8 | 130.978 | 93.88 | 0.92 |
| chr6_P1 | 365 | 517.94 | 1.4 | 10.24 | 96.43 | 0.97 |
| Total | 5,269 | 3,246.91 |  |  |  |  |
| Average |  | 541.15 | 0.84 |  |  |  |

#### Table S7 Description of the basic characteristics of the six linkage groups in females.

| Linkage/n Group ID | Total/n Marker | Total/n Distance (cM) | Average/n Distance (cM) | Max/n Gap (cM) | Gap/n<5 cM (%) | Spearman/n correlation coefficient |
| --- | --- | --- | --- | --- | --- | --- |
| chr1_P2 | 93.00 | 174.32 | 1.90 | 19.85 | 90.22 | -0.12 |
| chr2_P2 | 96.00 | 131.02 | 1.40 | 8.00 | 95.79 | 0.19 |
| chr3_P2 | 51.00 | 175.70 | 3.50 | 13.63 | 72.00 | 0.99 |
| chr4_P2 | 53.00 | 190.69 | 3.70 | 47.81 | 82.69 | 0.91 |
| chr5_P2 | 71.00 | 170.89 | 2.40 | 29.11 | 90.00 | 0.98 |
| chr6_P2 | 28.00 | 143.20 | 5.30 | 19.41 | 62.96 | 0.95 |
| Total | 392.00 | 985.82 |  |  |  |  |
| Average |  | 164.30 | 3.03 |  |  |  |

#### Table S8 Detailed information about the stable QTLs.

| Traits/ ID | QTL code | Linkage group | The number of QTLs | The number of QTL associated markers | The position on the genetic map | LOD threshold | Exp% |
| --- | --- | --- | --- | --- | --- | --- | --- |
| Epimedin C | *qEEC-4-1* | LG4 | 31 | 1 | 14.672-14.672 | 3.000 | 4.000 |
|  | *qEEC-4-2* | LG4 |  | 2 | 16.137-16.137 | 3.040 | 8.300 |
|  | *qEEC-4-3* | LG4 |  | 9 | 17.602-18.528 | 3.096 | 8.456 |
|  | *qEEC-4-4* | LG4 |  | 7 | 27.442-27.448 | 3.660 | 10.600 |
|  | *qEEC-4-5* | LG4 |  | 6 | 29.139-30.250 | 3.612 | 10.450 |
|  | *qEEC-4-6* | LG4 |  | 4 | 31.370-31.370 | 3.780 | 11.100 |
|  | *qEEC-4-7* | LG4 |  | 7 | 33.068-33.586 | 3.860 | 11.400 |
|  | *qEEC-4-8* | LG4 |  | 10 | 49.904-51.648 | 3.328 | 9.370 |
|  | *qEEC-4-9* | LG4 |  | 1 | 150.011-150.011 | 3.460 | 9.900 |
|  | *qEEC-4-10* | LG4 |  | 5 | 152.471-153.068 | 3.288 | 9.240 |
|  | *qEEC-4-11* | LG4 |  | 1 | 168.353-168.353 | 3.220 | 9.000 |
|  | *qEEC-4-12* | LG4 |  | 1 | 169.767-169.767 | 3.280 | 9.200 |
|  | *qEEC-4-13* | LG4 |  | 2 | 171.179-171.179 | 3.180 | 8.800 |
|  | *qEEC-4-14* | LG4 |  | 2 | 172.592-172.592 | 3.280 | 9.200 |
|  | *qEEC-6-1* | LG6 |  | 4 | 232.398-233.996 | 3.425 | 13.450 |
|  | *qEEC-6-2* | LG6 |  | 1 | 237.258-237.258 | 3.240 | 12.800 |
| Epimedin C | *qEEC-6-3* | LG6 |  | 1 | 243.390-243.390 | 3.640 | 14.300 |
|  | *qEEC-6-4* | LG6 |  | 2 | 245.583-245.583 | 3.100 | 12.300 |
|  | *qEEC-6-5* | LG6 |  | 3 | 248.800-250.710 | 3.323 | 13.100 |
|  | *qEEC-6-6* | LG6 |  | 1 | 251.990-251.990 | 3.620 | 14.200 |
|  | *qEEC-6-7* | LG6 |  | 2 | 264.122-264.122 | 4.235 | 16.400 |
|  | *qEEC-6-8* | LG6 |  | 3 | 265.401-265.401 | 4.150 | 16.100 |
|  | *qEEC-6-9* | LG6 |  | 6 | 266.681-269.075 | 4.155 | 16.083 |
|  | *qEEC-6-10* | LG6 |  | 1 | 271.491-271.491 | 4.110 | 15.900 |
|  | *qEEC-6-11* | LG6 |  | 1 | 277.921-277.921 | 4.270 | 16.500 |
|  | *qEEC-6-12* | LG6 |  | 2 | 282.280-282.866 | 3.360 | 13.200 |
|  | *qEEC-6-13* | LG6 |  | 4 | 287.301-288.358 | 4.295 | 16.600 |
|  | *qEEC-6-14* | LG6 |  | 5 | 290.136-293.353 | 3.816 | 14.880 |
|  | *qEEC-6-15* | LG6 |  | 7 | 295.023-296.785 | 3.903 | 15.186 |
|  | *qEEC-6-16* | LG6 |  | 1 | 297.951-297.951 | 4.350 | 16.800 |
|  | *qEEC-6-17* | LG6 |  | 1 | 309.572-309.572 | 3.940 | 15.300 |
| Total flavonoid content | *qETFC-5-1* | LG5 | 1 | 1 | 504.728-506.728 | 3.957 | 15.400 |
| Leaf area | *qELA-3-1* | LG3 | 2 | 1 | 210.022-210.022 | 3.670 | 16.800 |
|  | *qELA-5-1* | LG5 |  | 2 | 489.294-489.994 | 3.255 | 15.050 |
| Leaf length | *qELL-3-1* | LG3 | 12 | 1 | 210.022-210.022 | 3.680 | 16.800 |
|  | *qELL-5-1* | LG5 |  | 1 | 360.060-360.060 | 3.520 | 16.200 |
| Leaf length | *qELL-5-2* | LG5 |  | 1 | 362.640-362.640 | 3.550 | 16.300 |
|  | *qELL-5-3* | LG5 |  | 2 | 367.493-368.070 | 3.490 | 16.000 |
|  | *qELL-5-4* | LG5 |  | 1 | 373.305-373.305 | 3.270 | 15.100 |
|  | *qELL-5-5* | LG5 |  | 2 | 410.092-410.681 | 3.575 | 16.400 |
|  | *qELL-5-6* | LG5 |  | 3 | 412.481-413.070 | 3.743 | 17.100 |
|  | *qELL-5-7* | LG5 |  | 3 | 414.258-415.432 | 3.467 | 15.933 |
|  | *qELL-5-8* | LG5 |  | 2 | 418.466-419.05 | 3.760 | 17.150 |
|  | *qELL-5-9* | LG5 |  | 2 | 422.727-423.306 | 3.750 | 17.100 |
|  | *qELL-5-10* | LG5 |  | 5 | 424.476-424.476 | 3.804 | 17.340 |
|  | *qELL-5-11* | LG5 |  | 6 | 433.151-434.778 | 3.262 | 14.950 |
